# Traceable characterization of hollow organosilica beads as potential reference materials for extracellular vesicle measurements with optical techniques

**DOI:** 10.1186/s11671-024-03956-3

**Published:** 2024-01-22

**Authors:** Jérôme Deumer, Robin Schürmann, Anikó Gaál, Zoltán Varga, Britta Bettin, Edwin van der Pol, Rienk Nieuwland, David Ojeda, Aneta Sikora, Dorota Bartczak, Heidi Goenaga-Infante, Johanna Noireaux, Mahrad Khakpour, Virpi Korpelainen, Christian Gollwitzer

**Affiliations:** 1 Physikalisch-Technische Bundesanstalt, Abbestr. 2-12, 10587 Berlin, Germany; 2 Institute of Materials and Environmental Chemistry, Research Centre for Natural Sciences, Magyar Tudósok Körútja 2, Budapest, 1117 Hungary; 3 Department of Physical Chemistry and Materials Science, Budapest University of Technology and Economics, Műegyetem rkp. 3, Budapest, 1111 Hungary; 4 Laboratory of Experimental Clinical Chemistry, Amsterdam UMC Location University of Amsterdam, Meibergdreef 9, Amsterdam, The Netherlands; 5 Biomedical Engineering and Physics, Amsterdam UMC location University of Amsterdam, Meibergdreef 9, Amsterdam, The Netherlands; 6 Amsterdam Vesicle Center, Amsterdam UMC location University of Amsterdam, Meibergdreef 9, Amsterdam, The Netherlands; 7 National Measurement Laboratory, LGC Limited, Teddington, TW11 0LY UK; 8 Department of Climate Change and Environment, Laboratoire National de Métrologie et d’Essais, 1, Rue Gaston Boissier, 75724 Paris, France; 9 National Metrology Institute, VTT MIKES, Tekniikantie 1, FI-02150 Espoo, Finland

**Keywords:** Nanometrology, Extracellular vesicles, Flow cytometry, Particle tracking analysis, Reference materials, Hollow organosilica beads

## Abstract

The concentration of cell-type specific extracellular vesicles (EVs) is a promising biomarker for various diseases. However, concentrations of EVs measured by optical techniques such as flow cytometry (FCM) or particle tracking analysis (PTA)  in clinical practice are incomparable. To allow reliable and comparable concentration measurements suitable reference materials (RMs) and SI-traceable (SI—International system of units) methods are required. Hollow organosilica beads (HOBs) are promising RM candidates for concentration measurements of EVs based on light scattering, as the shape, low refractive index, and number concentration of HOBs are comparable to EVs of the respective size range that can be detected with current optical instrumentation. Here, we present traceable methods for measuring the particle size distribution of four HOB types in the size range between 200 and 500 nm by small-angle X-ray scattering (SAXS) and atomic force microscopy (AFM), as well as the number concentration by single-particle inductively coupled plasma mass spectrometry (spICP-MS). Based on the size and shape results, traceable reference values were obtained to additionally determine the refractive index of the shell of the HOB samples by FCM. Furthermore, the estimated refractive indexes of the HOBs plausibly agree with the refractive indexes of EVs of corresponding size. Due to their narrow size distribution and their similar shape, and low refractive index, all HOB samples studied are suitable RM candidates for calibration of the measured sample volume by optical methods within the photon wavelength range used, and thus for calibration of number concentration measurements of EVs in the size range indicated. This was confirmed as the number concentration values obtained by PTA and two independent flow cytometric measurements agreed with the concentration reference values obtained by two independent spICP-MS measurements within the calculated uncertainty limits.

## Introduction

Medical decisions require reliable information. Much of this information comes from the analysis of body fluids. Body fluids are readily available and provide information in real time. Liquid biopsy in this context refers to a minimally invasive method in which body fluids such as urine and plasma are collected and their (molecular) content is analyzed [1]. At present, there is growing interest in potentially clinically relevant information associated with extracellular vesicles (EVs) in liquid biopsies [2]. EVs are submicron cell-derived particles in body fluids and can be used as potential biomarkers for diseases such as cancer [3], inflammation [4] and cardiovascular disease [5]. The most widely studied body fluid for biomarker studies is human blood plasma [6, 7], which contains multiple types of spherical biological particles within a size range of $10 \, \textrm{nm}$ to $1 \, \upmu \textrm{m}$. EVs gained a lot of interest as they are released from all cell types and their biochemical composition, concentration and function are disease-dependent. Nevertheless, despite their potential as biomarkers, EV studies are limited in clinical settings, due to the lack of standardization of analytical methods [8]. EVs may differ in terms of biogenic mechanisms, composition, number concentration or morphology [9]. In addition, the separation and size measurement of EVs is challenging [10].

Generally, the size distribution or number concentration of EVs is determined by optical methods such as flow cytometry (FCM) or particle tracking analysis (PTA). A flow cytometer detects the scattered light and fluorescence signal of particles in a fluid stream, while PTA tracks the Brownian motion of individual particles within the sample volume by visualising the scattered light/fluorescence signal of the particles with a camera through a light microscope [11–13]. FCM and PTA are among the most commonly used clinical methods, as FCM allows high-throughput (thousands per second) measurement of individual EVs, while PTA allows simultaneous tracking of detected particles in the sample volume using dedicated PTA software [6, 10–12, 14].

Measurements of EV concentration with FCM have been shown to be inconsistent and therefore not comparable between different instruments because the EV population is heterogeneous in shape and size and consequently not all EVs reach the lower limit of detection (LoD), so only the upper fraction is detected in terms of particle diameter of the total particle population [15]. In the latest published inter-laboratory comparison study from 2018, of 46 flow cytometers evaluated, 31 could detect EVs $> 1200 \, \textrm{nm}$ in diameter, 21 could detect EVs $> 600 \, \textrm{nm}$ in diameter, and only 6 could detect EVs $> 300 \, \textrm{nm}$ in diameter by light scattering [16]. Today, some of the latest flow cytometers can detect EVs $> 100 \, \textrm{nm}$ in size, but they are not representative for most FCM.

An instrument similar to a flow cytometer is the nanoFCM, which uses a more focused laser beam and sample stream to detect particles in the size range of 50–$100 \, \textrm{nm}$ [10, 17], which covers the majority of EVs present in body fluids. However, in body fluids such as plasma, the proportion of lipoproteins increases steeply, making direct measurement of EVs with such instruments difficult [17, 18].

For modern FCM, the exact LoD in terms of particle size remains unknown because the scattering signals have arbitrary units, so the scattering signals cannot be directly related to EV diameter, which is generally a challenge. Therefore, to enable valid size-resolved concentration measurements of EVs, suitable reference materials (RM) are required, which are materials sufficiently stable and homogeneous with respect to one or more specified properties [19]. Similarly, PTA concentration measurements of polydisperse ensembles are strongly influenced by the size and optical properties of the measured particles as well as by the experimental setup (laser/camera settings).

To enable valid size-resolved concentration measurements of EVs, RMs with precisely defined shape, size, refractive index, and number concentration are needed to mimic the optical properties of EVs, as well as traceable techniques to characterize these RMs [12]. The term "traceable" in this context refers to metrological traceability to the International System of Units (SI), which means that the results of physical measurements can be referenced to SI units through an unbroken chain of comparisons, each with a stated uncertainty of measurement. Measurement results can therefore be quantified in absolute units within the limits of the stated uncertainties and compared across different instruments and physical measurement principles [20]. However, metrological traceability in most cases requires either a special measurement setup and careful data evaluation that takes into account the uncertainty contributions of all input quantities, or similar reference materials that can be used as a standard of comparison. Typical clinical measurement methods, such as FCM and PTA, are therefore not considered traceable methods per se﻿—however, through appropriate calibration with suitable RMs, these methods can provide accurate measurements that can be traced back to the RM.

Such RMs with traceably determined properties and number concentrations suitable for the calibration of state of the art FCM or PTA devices are not yet available. Possible RM candidates for FCM include liposomes, low refractive index (RI) nanoparticles, and hollow organosilica beads (HOBs) [10, 21]. HOBs with porous shells have attracted much attention recently, mainly because of their applicability as drug carriers.

In addition, the low RI of porous silica and the ability to form well-defined and, compared to liposomes, highly monodisperse hollow shell structures, even at diameters $> 100 \, \textrm{nm}$, make HOBs ideal candidates as scattering calibrators for optical measurements of EVs. Moreover, the adjustable shell thickness allows a tailored effective RI of the particle. The benefits of HOBs are especially true in direct comparison to conventional polystyrene or solid silica beads, whose RI is significantly higher than that of EVs [12].

A recent inter-laboratory comparison study on the number concentration of gold nanoparticles compared a wide range of optical methods, including PTA, inductively coupled single particle plasma mass spectrometry (spICP-MS), ultraviolet–visible (UV–Vis) light spectroscopy, centrifugal liquid sedimentation (CLS) and small- angle X-ray scattering (SAXS) [22]. With the exception of spICP-MS, which allows direct determination of the number concentration traceable to the unit kilogram by means of a dynamic mass flow (DMF) without reference nanomaterial, all methods require additional material parameters as input, such as. e.g.. particle mass density or RI value, to determine the number concentration. However, these parameters are hardly known for HOBs at the time of the present work.

In the present study, we prepared HOBs ranging in size from 50 nm to $450 \, \textrm{nm}$ and selected four HOB types to present traceable measurements of their number concentration. The diameter of these four HOB types ranges from approximately 200 nm to $500 \, \textrm{nm}$, representing the upper, non-negligible size range of EVs [12, 23], by spICP-MS. In addition, the specific size distribution of each type was traceably determined by AFM (particle counting method) and SAXS (ensemble method) [24]. The size range of HOBs was deliberately chosen to match the analytical measurement range of most modern flow cytometers, excluding nanoFCMs, to allow calibration for number concentration measurements, although the size of EVs present in body fluids also extends below the detection limit of most FCMs [16].

The overall objective of this work is to establish traceable reference values for the number concentration and size distribution and to determine the RI of four promising HOBs that are potential RMs for EV measurements by optical methods. The possibilities and limitations of the traceable and non-traceable methods presented here, which were applied to both size distribution and particle number concentration, are discussed. The measurement results and uncertainties of the non-traceable methods are verified by comparison with the results of the traceable methods.

## Synthesis of HOBs

### Materials

1,2-Bis(triethoxysilyl)ethane (BTEE) ($96\%$), tetraethyl orthosilicate (TEOS, $\ge 99.0 \%$ GC), cyclohexane ($\ge 99.9 \%$), and L-arginine (reagent grade, $\ge 98\%$, TLC) were obtained from Sigma-Aldrich. Ethyl alcohol (EtOH, anhydrous, $>99.7\%$, HiPerSolv CHROMANORM for HPLC,—Gradient Grade) and ammonia solution (NH$_{4}$OH, $25\%$, AnalaR NORMAPUR) were purchased from VWR Chemicals. High-purity deionized water ($18.2\, \textrm{M}\Omega \, \textrm{cm}^{-1}$, Milli-Q (MQ), Merck) was used during the synthesis.

### Synthesis of core silica particles

SNP082 core silica particles were prepared in-house using the Stöber-method [25]. First, $250 \, \textrm{mL}$ EtOH, $25.3 \, \textrm{mL}$ ammonia, and $11.25 \, \textrm{mL}$ MQ water were mixed at 350 rpm in a $500 \, \textrm{mL}$ laboratory bottle from borosilicate glass (VWR) using a magnetic stirrer bar (25 mm $\times$ 6 mm). Then, $7.5 \, \textrm{mL}$ TEOS was added to the solution drop-wise with a serological pipette ($10 \, \textrm{mL}$; VWR) using a pipettor with an average speed of $15 \, \textrm{mL} \, \textrm{min}^{-1}$. Afterward, the reaction mixture was stirred at 350 rpm overnight at ambient conditions ($25 ^{\circ }\textrm{C}$, 1 atm). The particles were washed three times with EtOH using centrifugation at $7000 \, \times \, \textrm{g}$ (Eppendorf Centrifuge 5804 R with FA-45-6-30 rotor, Eppendorf Austria GmbH Wien, Austria). The final SNP082 particles were dispersed in $45 \, \textrm{mL}$ water.

### Preparation of HOBs

**Fig. 1:**
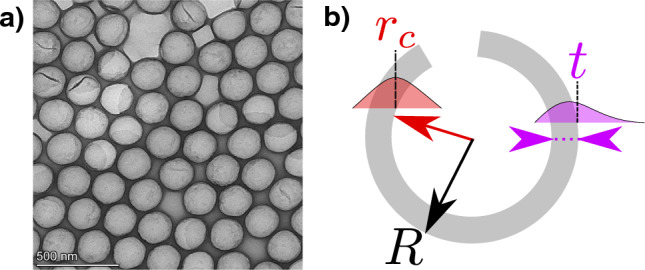
**a** Transmission electron microscopy (TEM) image of the 02-HOB-AN200 sample. **b** Schematic representation (not true to scale/proportion) of a hollow organosilica bead (HOB) particle with model parameters: $r_c$-log-normal distributed core radius, *t*-Log-normal distributed shell thickness, *R*-total radius. The HOB shell is not completely closed to allow the etching of the core

HOB samples were prepared using a hard template method combined with a basic amino acid catalysis route, as described previously [26]. $200 \, \textrm{nm}$, and $400 \, \textrm{nm}$ nominal diameter silica core particles from Alpha Nanotech (Vancouver, Canada) were used for the preparation of 02-HOB-AN200, as shown in Fig. 1, and 11-HOB-AN400 samples in $20 \, \textrm{mL}$ glass vials (Wheaton Industries, New Jersey, US), while $400 \, \textrm{nm}$ nominal diameter silica core particles (PSI$-$0.4) from Kisker Biotech (Steinfurt, Germany) and the previously described SNP082 particles were used to prepare 09-HOB-K400-05 and 04-HOB-SNP082 samples in 100 mL glass vials (VWR), respectively. All reaction parameters are summarized in Table 1. After the etching and washing steps, HOBs were dispersed in water in volumes corresponding to the volume of the aqueous phase during the synthesis of the core-shell particles for each sample.

**Table 1:** Reaction parameters for preparing different hollow organosilica beads (HOBs)

Sample	Glass vial /mL	Magnetic stirrer bar size /$\textrm{mm} \times \textrm{mm}$	Stirring speed /rpm	Solid Content of the seed particle samples /$\textrm{mg} \, \textrm{mL}^{-1}$	l-arginine (dry weight/volume of stock solution) /$\textrm{mg} \, \textrm{mL}^{-1}$	Seed particle volume /$\upmu \textrm{L}$	MQ /$\textrm{mL}$	Cyclohexan /$\upmu \textrm{L}$	BTEE /$\upmu \textrm{L}$	Centrif. speed / $\times \textrm{g}$	Etching time /h
02-HOB-AN200	20	$10 \times 2$	300	50	26	1500	8	650	670	8000	22
04-HOB-SNP082	100	$20 \times 2$	300	39.5	$\frac{78}{11}$	9000	40	3900	4020	6000	10
09-HOB-K400-05	100	$10\times 2$	300	50	$\frac{78}{8.55}$	11450	40	3900	4020	5000	12
11-HOB-AN400	20	$20 \times 2$	300	50	26	1500	8	650	670	5000	20

BTEE: N-Benzoyl-L-tyrosine ethyl ester

## Measurement methods

### Traceable methods

#### AFM

The Jupiter XR AFM was used for the AFM measurements on the particles. A tapping mode and standard silicon tips were used for the measurements. The *z*-scale of the AFM was calibrated with step height standards calibrated with the MIKES metrological AFM (MAFM) and directly traceable to the metre via an online interferometric position measurement [27]. Particles were deposited on a poly-L-lysine treated MICA surface.

**Fig. 2:**
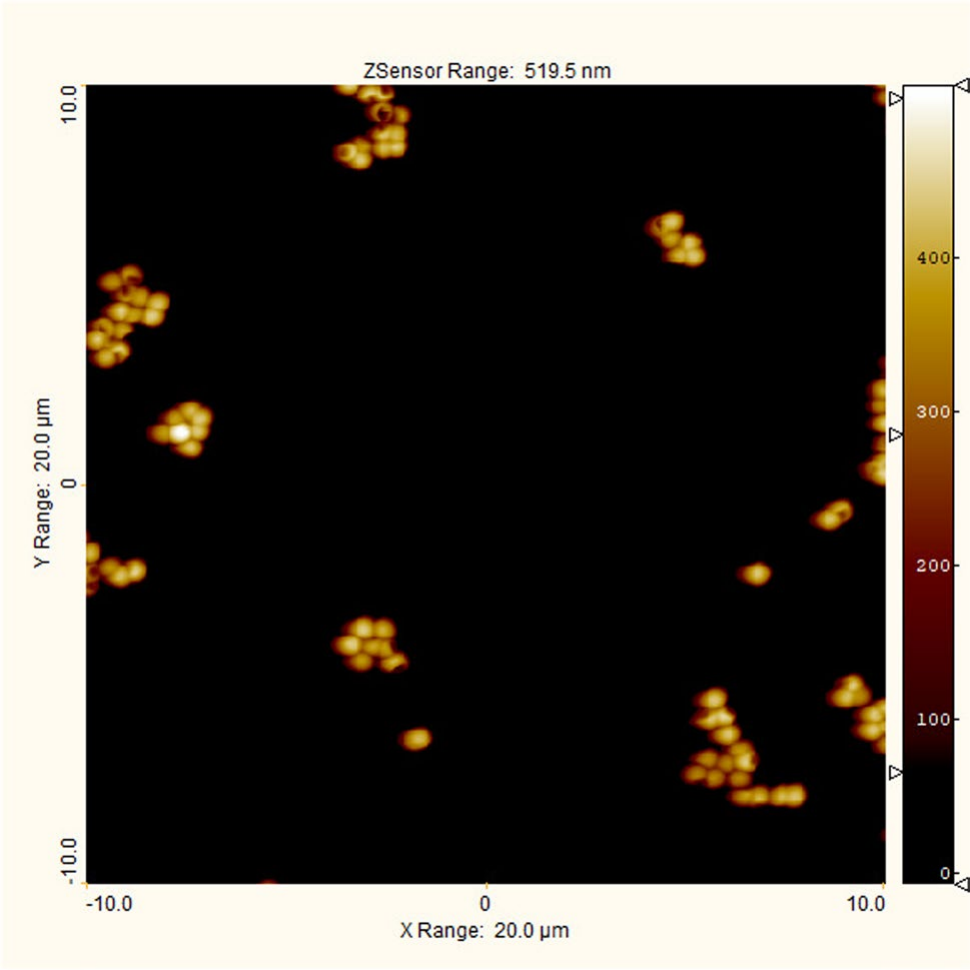
Atomic force microscope (AFM) image of sample 11-HOB-AN400 with a size of $20 \, \upmu \textrm{m} \times 20 \, \upmu \textrm{m}$. Individual particles, agglomerated particles, and layers of particles can be seen. Particle height was analyzed only on individual particles. Agglomerated particles were excluded from the analysis

For AFM measurements, the particle height can directly be measured. The measurement ranges were chosen so that the individual particles could be clearly distinguished from each other on the AFM image. To determine the size distribution, a series of $\sim 500$ single particles was measured for each particle type. Sample tilt and/or substrate flatness error were corrected prior to height measurements. The Maugis and Pollock method was used to correct for particle deformation caused by surface forces [28].

The total expanded uncertainty *U* of the mean diameter *d* of the HOBs was determined by considering an expanded systematic measurement uncertainty of $\pm 7.2 \, \textrm{nm}$ ($k=2$) plus a much smaller contribution due to the expanded statistical uncertainty of the mean ($k=2$). The main uncertainty components are instrument *z*-scale calibration, surface roughness and uncertainty caused by the analysis method including the substrate flatness correction. System non-linearity and particle deformation has a minor effect on the uncertainty. Since both uncertainty budgets are not correlated, Gaussian uncertainty propagation was used to determine *U*(*d*).

#### SAXS

SAXS is a widely proven, non-destructive measurement method for characterizing ensembles of suspended nanoparticles [29–33].

**Fig. 3:**
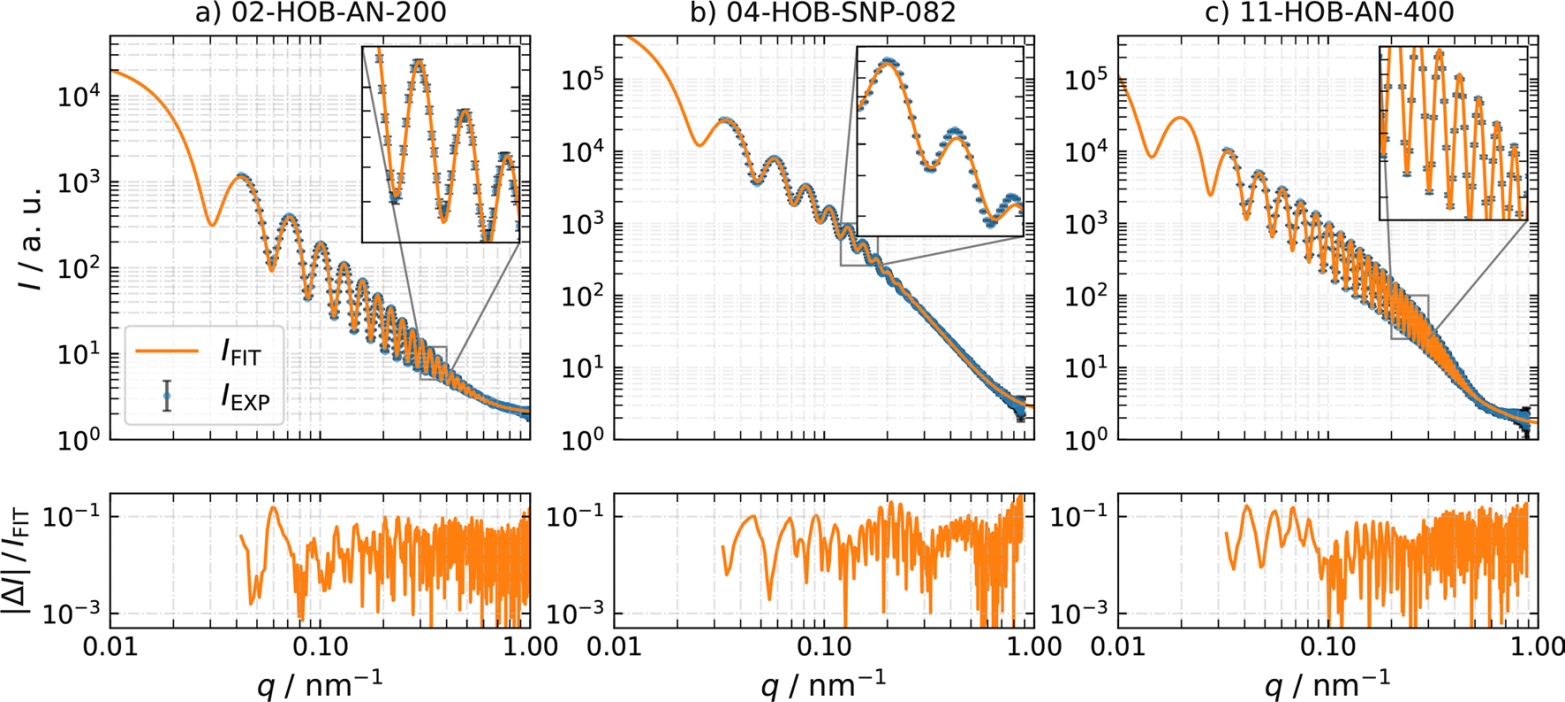
Small-angle X-ray scattering (SAXS) results. The experimental data of all samples $I_{\textrm{EXP}}$ shows pronounced oscillations. The relative residuals of the model function $I_{\textrm{FIT}}$ compared to $I_{\textrm{EXP}}$ are shown in the bottom plots. Table 3 contains all the results from the SAXS measurements

To obtain Information about the particle ensemble, e.g. the size distribution [32], the 1D scattering curve was fitted with a hollow sphere model (Eqs. 1–3) with the following fit parameters: $r_{\textrm{c}}$—the core radius, $\sigma _{\textrm{c}}$—standard deviation of the underlying log-normal size distribution of $r_{\textrm{c}}$, *t*—the shell thickness, $\sigma _{\textrm{t}}$—the standard deviation of the log-normally distributed shell thickness, $\Delta \rho _{\textrm{c}}$/$\Delta \rho _{\textrm{s}}$—the electron density contrast ratio of the core compared to the shell, the eccentricity of the core, $\sigma _{\textrm{q}}$—the width of *q*-smearing, and $c_0$—the constant background scattering. The corresponding scattering intensity for dilute liquid suspensions as a function of the momentum transfer *q* of the photons is obtained by convolving the absolute square of the form factor $F_{\textrm{hc}}$ (hc: hollow sphere, see Fig. 3b, but without the hole) of the particles with $G(r_c)$ and *L*(*t*), which describe the Gaussian size distributions of the radius of the concentric core $r_{\textrm{c}}$ and the log-normally distributed shell thickness *t*, respectively:

1 $$\begin{aligned} I(q) \propto \int \limits _0^\infty \int \limits _0^\infty \mid F_{\textrm{hc}}(q, r_{\textrm{c}}, t, \Delta \rho _{\textrm{c}}, \Delta \rho _{\textrm{s}}) \mid ^2 \, G(r_{\textrm{c}}) \, L(t) \, \textrm{d}r_{\textrm{c}} \textrm{d}t + c_0, \end{aligned}$$

where $F_{\textrm{hc}}$ can be constructed from spherical form factors $F_{\textrm{sph}}$ [34], such that

2 $$\begin{aligned} F_{\textrm{hc}}(q) = F_{\textrm{sph}}(q, r_{\textrm{c}} + t, \Delta \rho _{\textrm{s}}) - F_{\textrm{sph}}(q, r_{\textrm{c}}, \Delta \rho _{\textrm{c}}). \end{aligned}$$

$r_{\textrm{c}}$ and *t* are completely decoupled, so that for the total particle radius $R = r_{\textrm{c}} + t$ holds. To account for any beam smearing effects on the theoretical scattering curve *I*(*q*) with infinitesimally small beam size, *I*(*q*) must again be convolved with a Gaussian distributed resolution function $\Theta$, i.e., a broadening of *I*(*q*) in *q* such that [35]

3 $$\begin{aligned} I_{\textrm{bm}}(q) = \int \limits _{0}^\infty \Theta (q - z) \, I(z) \, \textrm{d}z. \end{aligned}$$

In order to fit the experimental data within a reasonable amount of time, equation 3 was calculated using a Monte Carlo approach. This involved drawing 2000 samples for each parameter $r_{\textrm{c}}$, *t*, and *z* corresponding to each specific distribution to calculate $I_{\textrm{bm}}(q)$. For ﻿each sample, the corresponding scattering curve was analysed using software developed in-house.

In the uncertainty-weighted least squares method [36, 37], the experimental data were first fitted to obtain the best fit parameters, and then, starting from the best fit value of the core radius as well as the shell thickness, an independent uncertainty scan of both variables was performed to obtain information about their mean value as well as uncertainty *u*. The uncertainty u ($k = 1$) was estimated from the confidence interval where the reduced $\chi ^2$ does not exceed twice the minimum of the best fit $\chi _{\textrm{min}}^2$. The expanded uncertainty *U* ($k = 2$) was determined by $U = 2 \, u$.

In the following, the *particle size* refers to the diameter *d* of the hollow sphere particles with $d = 2 R$, where $R = r_{\textrm{c}} + t$ is the total particle radius. In addition, the uncertainty *u*(*R*) was determined by Gaussian uncertainty propagation, since the uncertainties $u(r_{\textrm{c}})$ and $u(r_{\textrm{t}})$ are not correlated. Moreover, $\sigma _{\textrm{R}}$ was determined by Monte-Carlo-sampling of $(\sigma _{\textrm{c}} + \sigma _{\textrm{t}})$.

All SAXS measurements were carried out at the four-crystal monochromator beamline of the Physikalisch-Technische Bundesanstalt at the BESSY II synchrotron radiation facility in Berlin-Adlershof using the SAXS facility of the Helmholtz-Zentrum Berlin.

For sample preparation, each colloidal solution was filled into separate rectangular capillaries (*Hilgenberg GmbH, Germany*) made of borosilicate glass with uniform thickness along the vertical axis and sealed with a welding torch before measurement. The filled capillaries were then loaded into the vacuum chamber of the SAXS facility and irradiated with a beam of synchrotron radiation with an energy of 8 keV and a cross-sectional area of 150 $\times$ 400 $\mathrm {\upmu m}^2$ at the position of the capillaries. During the measurement, the capillaries are scanned vertically with the pencil beam and measured accordingly at predetermined vertical positions. The radiation scattered by the samples is then recorded by a vacuum-compatible Pilatus 1M detector [38], which is located between 3 m and 5 m behind the sample holder. Prior to data analysis, azimuthal integration of the scattering image is performed using proprietary software. For the background correction, another capillary filled with the suspension medium (usually water) only is measured in addition to the actual samples. This background scattering curve is subtracted from the measured scattering curves of the samples, normalized to the respective thickness of the capillaries, before data evaluation.

#### spICP-MS

spICPM-MS was performed at two different laboratories.

At Laboratoire National de Metrologie et d’Essais (LNE), the analysis was performed using a sector field ICP-MS (Thermo Element XR) in medium resolution mode with a dwell time of 3 ms in analog mode for the isotope $^{28}$Si. The instrument was equipped with a seaspray nebulizer resulting in an uptake rate of about $200 \, \upmu \textrm{L} \, \textrm{min}^{-1}$ and a Scott double pass spray chamber cooled to $3 ^{\circ }$C. At least 40,000 scans with a total duration of 2 min were performed in each run to detect at least 2000 particles. To reduce the background contribution to the signal caused by the glassware, a sapphire injector was used instead of the standard quartz injector. The instrument was tuned daily and the transport coefficient was calculated based on the size method [39] using two gold nanoparticle suspensions of $40 \, \textrm{nm}$ and $60 \, \textrm{nm}$ and a series of dissolved gold solutions in the range from $0.05 \, \mathrm {ng/g}$ to $2 \, \mathrm {ng/g}$. The correlation coefficients of both the dissolved and particulate standards were above 0.99. The calibration standards were analyzed at the beginning and end of each series of measurements, and the variation in transport efficiency between the beginning and end of the series was less than $10\,\%$. Particle concentrations were determined as follows after diluting the original suspensions in water:

4 $$\begin{aligned} C = \frac{N}{\eta _{\textrm{size}} \cdot V}, \end{aligned}$$

where *N* is the number of particles detected in the time scan, $\eta _{\textrm{size}}$ is the transport efficiency, and *V* is the sample mass flow rate.

The transport efficiency was calculated based on the size method with the following equation:

5 $$\begin{aligned} \eta _{\textrm{size}} = \frac{K}{V\cdot t_{\textrm{dwell}} \cdot R_{\textrm{L}}}, \end{aligned}$$

where *K* is the linear regression of intensity versus the gold ionic concentration of the standards, $R_{\textrm{L}}$ is the linear regression of mean particle intensity versus the mean mass of the gold particle standards, $t_{\textrm{dwell}}$ is the dwell time chose for the analysis.

Three different suspensions were prepared on three different days and the results were combined to give the final particle concentrations. All calculations were performed with in-house Excel spreadsheets.

At National Measurement Laboratory (LGC), spICP-MS measurements were performed using an Agilent Technologies 8900 ICP-MS instrument. The instrument was equipped with a micromist nebulizer with a pump rate of 0.1 rpm, a Scott double pass spray chamber cooled to 2$^\circ$ C, MassHunter 4.4 software (version: G72dC C.01.03), and a microsecond detection function allowing analysis in single particle mode. The instrument was tuned to obtain the best signal-to-noise ratio daily with a 1 $\upmu \textrm{g} \, \textrm{L}^{-1}$ Agilent tuning solution containing Li, Y, and Tl to verify instrument performance. Subsequently, the instrument response factor was optimized to $^{28}$Si with 1 $\upmu \textrm{g} \, \textrm{kg}^{-1}$ (gravimetrically diluted with 1 mM Na$_{3}$Ct) elemental Si standard (Romil) to achieve the best instrument sensitivity with minimal background contribution. spICP-MS analysis in rapid transient analysis (TRA) mode was performed with a dwell time of 100 $\upmu$s per point with no settling time between measurements and using the single particle application module of the ICP-MS MassHunter software (G5714A). Hydrogen was used throughout at a flow rate of 2 $\textrm{mL} \, \textrm{min}^{-1}$ to minimize interference. Both the Single Particle Application Module of the ICP-MS MassHunter software (G5714A) and Excel spreadsheets developed in-house were used for data processing. The instrument was cleaned with 1 mM Na$_{3}$Ct after each preparation. NanoXact, $200 \, \textrm{nm}$ silica particles from nanoComposix, characterized in-house, were used as quality control material. Three independent preparations of each sample were measured 5 times under repeatability conditions. Transport efficiency was determined using the DMF method, which provides direct traceability to the SI unit kilogram [40]. The particle concentration in the sample was determined using equation 4. The associated measurement uncertainty was calculated according to ISO 17025 and Eurachem/CITAC guidelines. For spICP-MS, the particle size, i.e. diameter, can only be calculated indirectly from the measured particle mass if the particle density, stoichiometry and shape are known. Such analyses/calculations were not performed. The size of the particles was not determined with spICP-MS because the technique measures element mass fraction (i.e. mass of silicon), rather than the size. Only if particle geometry, exact composition and density and very well characterised by other techniques, the size can be derived from the element mass fraction.

### Non-traceable methods

#### FCM

FCM measurements were performed with two different flow cytometers (A60-Micro, Apogee, UK and Nothern Lights, Cytek, US) in the Amsterdam University Medical Center, location AMC. For both flow cytometers, 02-HOB-AN200 was diluted 30,000-fold, 04-HOB-SNP082 40,000-fold, 09-HOB-K400-05 30,000-fold, and 11-HOB-AN400 2,000-fold in purified water (MQ at $20 ^{\circ }\textrm{C}$ with assumed mass density of $0.99984 \, \textrm{g} \, \textrm{mL}^{-1}$) and measured for $120 \, \textrm{s}$. For the A60-Micro, the flow rate $Q = 3.01 \, \upmu \textrm{L} \, \textrm{min}^{-1}$ was controlled with a syringe pump. The trigger was set on the side scattering detector with a threshold of 24 arbitrary units, which corresponds to a side scattering cross section of $\sim 8 \, \textrm{nm}^2$. The side scattering detector is a photomultiplier tube operating at 375 volt and gain 1, and detects scattered light with a wavelength of $405 \, \textrm{nm}$. Other detectors of the flow cytometer were not used in this experiment.

For the Northern Lights, the adjusted flow rate was $Q = 20 \, \upmu \textrm{L} \, \textrm{min}^{-1}$ whereas the actual flow rate and total sample volume were measured with a temperature-based flow rate sensor. The trigger was set on the side scattering detector with a threshold of 1500 arbitrary units, which corresponds to a side scattering cross section of $\sim 2 \, \textrm{nm}^2$. The side scattering detector is an avalanche photon detector operating at a gain of 2000, and detects scattered light with a wavelength of $405 \, \textrm{nm}$. Other detectors of the flow cytometer were not used in this experiment.

For the Northern Light, of which the light collection angles are well-specified, the measured side scattered light in arbitrary units was related to the theoretical scattering cross section of polystyrene beads in $\textrm{nm}^2$ using Rosetta Calibration (Exometry, The Netherlands) [41]. Rosetta Calibration contains a kit with a mixture of NIST-traceable polystyrene beads and software utilizing Mie theory based on the code of Mätzler [42]. Rosetta Calibration was used by following the instructions of the manufacturer. Based on this calibration, the reference values of the diameter of HOBs determined with AFM and SAXS, and the thicknesses of the shell of the HOBs, the refractive indices (RIs) of the shell of the HOBs were solved by least square fitting the median side scattering cross sections of three beads with MATLAB (v. 2020b, USA). Here, it was assumed that the core of the HOBs are filled with water having a RI = 1.3431. In addition, EVs were modelled as concentric particles having a shell with RI = 1.48 and a thickness of $6 \, \textrm{nm}$, and a core with a RI ranging from 1.35 to 1.40 [43]. The concentration *C* of HOBs was obtained by solving the following equation:

6 $$\begin{aligned} C = \frac{(N_1 + 2 \, N_2 + 3 \, N_3) \cdot D}{Q \cdot t_{\textrm{MEAS}} \cdot \rho }, \end{aligned}$$

where $N_1$, $N_2$, and $N_3$ are the number of counted singlets, doublets, and triplets of HOBs, respectively, *D* is the volumetric dilution factor, $t_{\textrm{MEAS}}$ is the measuring time, and $\rho$ is the density of water.

#### PTA

PTA is a well-known technique for particle size analysis [44]. PTA (NS300 instrument, Malvern Panalytical, equipped with a $405 \, \textrm{nm}$ diode laser source, sCMOS camera, syringe pump, and NTA3.4 software) was used to support size and concentration measurements on silica-based materials. All standards and samples were diluted in $1 \, \textrm{mM}$ Na$_{3}$Ct. Each sample was measured at least ten times under repeatability conditions. Quality control particle material, LGCQC5050 (www.lgcstandards.com; colloidal gold nanoparticles with a nominal diameter of 30 nm) was measured within the same measurement batch for quality control purposes with regards to size and number-based concentration. Material was found monomodal and monodispersed, within agreement with the certified values. The PTA instrument was turned on at least 30 min before measurements. All measurements were performed at room temperature. Videos were recorded over a 60-second period, with a 10-s equilibration period before each measurement. Camera focus was adjusted manually, the brightness (camera level) was set to 13 or 14 depending on the sample. Measurements were made in flow mode with the syringe pump set to an injection speed of 40. The software assumed the viscosity of water. For analysis of the recorded films, the detection threshold was set to 5. Three independent preparations of each sample were measured 5 times under repeatability conditions. For most samples, 15 measurements were performed, although for some aliquots the number of runs was between 10 and 15 for technical reasons, such as the detection of large dust particles. Nanoxact, $200 \, \textrm{nm}$ silica particles from nanoComposix, which were characterized internally, served as quality control material. The particle size was calculated by the software directly from the mean square displacement, the temperature and the assumed viscosity of water using the Stokes–Einstein equation, while the particle number concentration was calculated by multiplying the particle number mL$^{-1}$ given by the software by the sample dilution factor (gravimetric) and assuming a water density of $1 \, \textrm{g} \, \textrm{cm}^{-3}$. In general, measured particle number concentration depends on the instrument’s sensing volume, which is typically calibrated by the instrument’s manufacturer. The associated measurement uncertainty was calculated according to ISO 17025 and Eurachem/CITAC guidelines.

### Final reference values

The traceable measurement of the mean size, i.e., diameter, and size distribution of the HOBs was determined using SAXS and AFM. Since the two measurement methods are not correlated, the uncertainties of both methods were combined using Gaussian uncertainty propagation to obtain the corresponding reference value. For the particles with inconsistent values for the mean and uncertainty, the Birge ratio method [45] was used to combine the inconsistent values.

There are no uncertainties for the width of the size distribution of the individual values. Since the size distribution determined by AFM is probably more unique and thus reliable then the one determined by SAXS, these values of the size distribution widths were reported instead.

For traceability, spICP-MS was performed to determine the number concentration. For spICP-MS, it was also assumed that the two results ("size method" vs. DMF) were completely uncorrelated. Both results were combined using the Gaussian uncertainty propagation or Birge ratio method. For all reference values obtained in a traceable manner, PTA was used to confirm these results.

## Results and discussion

This section contains the measured size distribution, refractive index and number concentration of the samples described in section 3.

### AFM

The AFM images (Fig. 2) show that the particles are arranged in many different orientations on the surface. In the measurements of the 02-HOB-AN200 particles we observed a lot of small particles (size $< 10 \, \textrm{nm}$) but those particles were not included in the analysis and they didn’t affect the AFM results. The small particles in the other samples were not observed. In the figure, the differences in particle height can be seen, but also the different orientations of the particles.

Table 2 lists the diameter, standard deviation, and uncertainty of the diameter of the HOB samples measured by AFM.

**Table 2:** Results of hollow organosilica beads (HOB) by atomic force microscopy (AFM)

Sample	*d* / nm	$\sigma _{d}$/ nm	*U*(*d*) / nm
02-HOB-AN200	213.3	4.3	7.3
04-HOB-SNP082	257.7	10.2	7.4
09-HOB-K400-05	399.8	45.1	7.6
11-HOB-AN400	432.2	27.5	7.6

Results are for particle size, i.e., mean diameter *d*. *U*(*d*) is the expanded uncertainty ($k=2$) and $\sigma _{d}$ is the standard deviation of the underlying Gaussian size distribution

### SAXS

SAXS was used to determine the size distribution of all samples. All HOB-samples, except 09-HOB-K400-05, show clear oscillations in the experimental scattering curve $I_{\textrm{EXP}}$, which agree with our model function $I_{\textrm{FIT}}$, as indicated by low relative residuals of $\vert \Delta I \vert \, \mathrm {/} \, I_{\textrm{FIT}}$ and low $\chi _{\textrm{min}}^2$ values in Fig. 3 and Table 3, respectively. The good agreement of $I_{\textrm{FIT}}$ with $I_{\textrm{EXP}}$ is especially true for the minima of $I_{\textrm{EXP}}$, which are less pronounced due to beam smearing effects because of the relatively large particle size and the finite cross-sectional size of the X-ray beam. 09-HOB-K400 could not be evaluated with satisfactory validity with respect to its size distribution due to missing but necessary oscillations. For the 02-HOB-AN200 and 04-HOB-SNP082 the mean of the shell-thickness can be determined only with a large uncertainty. The reason for these large uncertainties in shell thickness is not easy to determine. However, possible causes could simply be the large number of parameters, so that when the specific fitting algorithm (Nelder-Mead) is used, various local minima are found in the remaining parameter space, which distort the uncertainty scan. Likewise, it cannot be ruled out that any statistical correlations between the parameters have an influence on the fitting algorithm. Nevertheless, the uncertainties define an upper boundary for the shell-thickness, which can be used for estimations of the RI.

Table 3 lists the mean, standard deviation, and standard uncertainty of the diameter *d* of the HOB samples measured by SAXS.

**Table 3:** Results of the small-angle X-ray scattering (SAXS) measurements

Sample	$r_{\textrm{c}}$ /nm	$U(r_{\textrm{c}})$ /nm	*t* /nm	*U*(*t*) /nm	*d* /nm	*U*(*d*) /nm	$\sigma _{d}$ /nm	$\chi _{\textrm{min}}^2$ /1
02-HOB-AN200	105.5	1.9	4.2	3.6	219	9	5	3.09
04-HOB-SNP082	126.6	5.7	5.1	5.8	264	17	12	25.05
09-HOB-K400-05								
11-HOB-AN400	228.5	1.6	8.8	1.5	475	5	5	6.2

*U*(*x*) is the expanded uncertainty ($k=2$) of the variable *x*. The particle size, i.e., diameter *d*, was calculated by multiplying the results for the total HOB radius $R = r_c + t$ and its uncertainty *U*(*R*) by two. $r_c$ is the mean core radius, *t* is the mean shell thickness, $\chi _{\textrm{min}}^2$ is the minimum of the least squares method and describes the quality of the fit

### spICP-MS

**Fig. 4:**
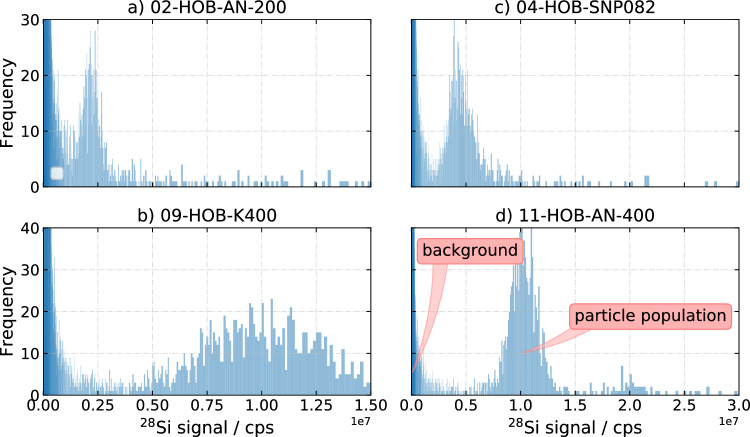
spICP-MS signal distribution from the National Measurement Laboratory (LGC). "cps" means counts per second. **a**/**c** Monomodal, monodispersed and well-resolved from the background. **b** Monomodal but of higher polydispersity, well-resolved from the background. **d** Well-resolved from the background, but multimodal

With HR-ICP-MS and millisecond residence time, only the largest HOBs, i.e. 09-HOB-K400-05 and 11-HOB-AN400, were analyzed due to the dominant silicon background signal, as depicted in Fig. 4. Table 4 shows the spICP-MS concentration of HOB samples measured by LNE using HR-ICP-MS with the "Size method" and by LGC using ICP/MS/MS with the DMF method.

**Table 4:** Number concentration from the single-particle inductively coupled plasma mass spectrometry (spICP-MS) measurements

	HR-ICP-MS ("Size method")		ICP-QQQ-MS (DMF)	
Sample	*C* / $\textrm{kg}^{-1}$	*U*(*C*) / $\textrm{kg}^{-1}$	*C* / $\textrm{kg}^{-1}$	*U*(*C*) / $\textrm{kg}^{-1}$
02-HOB-AN200			$3.88 \times 10^{14}$	$0.54 \times 10^{14}$
04-HOB-SNP082			$2.25 \times 10^{14}$	$0.32 \times 10^{14}$
09-HOB-K400-05	$8.94 \times 10^{13}$	$1.80 \times 10^{13}$	$8.29 \times 10^{13}$	$0.74 \times 10^{13}$
11-HOB-AN400	$5.80 \times 10^{13}$	$0.43 \times 10^{13}$	$4.34 \times 10^{13}$	$0.43 \times 10^{13}$

*C* is the mean number concentration, where *U*(*C*) is the expanded uncertainty (k=2)

### FCM

**Fig. 5:**
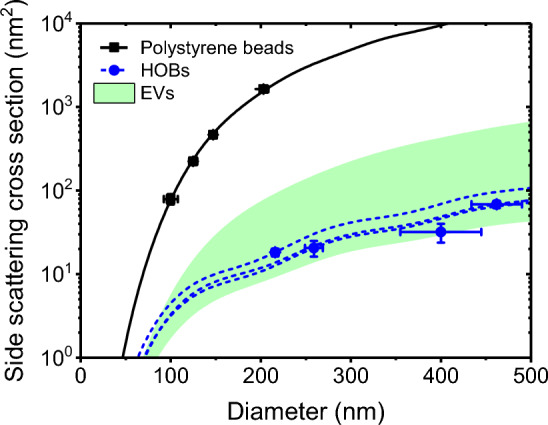
Side scattering cross section measured with a Cytek Northern Lights flow cytometer (symbols) and calculated with Mie theory (lines) versus the diameter of polystyrene beads (squares, solid lines) and hollow organosilica beads (HOBs; circles, dashed lines). The refractive index of polystyrene beads was 1.6328. The coefficient of determination $R^2$ of the fit is 0.9949 for polystyrene beads. The side scattering cross sections of three HOBs were fitted with Mie theory to resolve the refractive index of the shells. The dashed lines differ because the refractive index as well as the thicknesses of the shells differ. Extracellular vesicles (EVs) were modelled as concentric particles having a $6 \, \textrm{nm}$ thick shell with a refractive index of 1.48 and a core with a refractive index ranging from 1.35 to 1.40 (marked area)

Figure 5 shows the side scattering cross-section of polystyrene beads and HOBs measured by FCM (Cytek Northern Lights) compared to the determined reference values for diameter. With a coefficient of determination $R^2 = 0.9949$ for polystyrene beads, the theory describes the data well [46]. Compared to polystyrene beads, the side scattering cross sections of HOBs are about 2 orders of magnitude smaller, thus within the same order of magnitude as EVs.

Table 5 shows the measured concentration values as well as the calculated RI of the HOB shell for the corresponding shell thicknesses from SAXS using the mean particle size determined by SAXS and atomic force microscopy. For 02-HOB-AN200, the upper limit of the RI values appear to be too high, which could indicate that the lower limit of shell thickness determined by SAXS is underestimated. For 04-HOB-SNP082, no lower limit for shell thickness could be given due to its negative value, so only a lower limit for the RI value of 1.47 can be given. With a value between 1.48 and 1.53, the shell RI of 11-HOB-AN400 appears plausible. Realistic RI values were obtained for 04-HOB and 11-HOB, especially when the maximum shell thickness was selected. The effective RI, i.e., the RI averaged over the volume of a homogeneous particle of the same size, was also calculated for 11-HOB and ranges from 1.363 to 1.373 which matches the mean effective RI of EVs in human urine [47].

**Table 5:** Results of flow cytometry (FCM) of number concentration and shell refractive index. For concentration values, the first column refers to the Apogee A60-Micro measurement, while the second column refers to the Cytek Northern Lights measurement

Sample	*C* /$\textrm{kg}^{-1}$	*C* /$\textrm{kg}^{-1}$	shell refractive index	$t_{\textrm{FCM}}$ / nm
02-HOB-AN200	$3.46$ $\times 10^{14}$	$3.89$ $\times 10^{14}$	(1.54, 1.7, $\cdot$)	(7.8, 4.2, $\cdot$)
04-HOB-SNP082	$2.28$ $\times 10^{14}$	$1.9$ $\times 10^{14}$	(1.47, 1.60, $\cdot$ )	(10.9, 5.1, $\cdot$ )
09-HOB-K400-05	$8.15$ $\times 10^{13}$	$8.85$ $\times 10^{13}$		
11-HOB-AN400	$8.33$ $\times 10^{13}$	$5.19$ $\times 10^{13}$	(1.48, 1.50, 1.53)	(10.3, 8.8, 7.3)

The FCM results also include estimated values for the shell refractive index (lower limit (lb), mean, upper limit (ub)) of the organosilica hollow spheres based on scattering cross sections measured with the Cytek Northern Light, mean diameters measured with small-angle X-ray scattering (SAXS) and atomic force microscopy (AFM), and shell thicknesses $t_{\textrm{FCM}}$ (ub, mean, lb) measured with SAXS. The calculated refractive indices belong to the corresponding $t_{\textrm{FCM}}$ (lb, mean, ub), while no lower $t_{\textrm{FCM}}$ was available for 04-HOB-SNP082 due to its negative value. Also for 02-HOB-AN200, a shell thickness of $0.6 \, \textrm{nm}$ is implausible, so the corresponding refractive index is not given. For 09-HOB-K400-05, the refractive index could not be determined either, as the results of the small-angle X-ray scattering (SAXS) were not available

### PTA

The number concentration and diameter of the HOBs measured with PTA are presented in Table 6. During each series of measurements, NanoXact 200 nm SiO2 from NanoComposix was measured as an additional control sample. The test material proved to be monomodal and monodisperse, size and concentration were in agreement with the specifications of the manufacturer as well as with the measurements carried out internally using spICP-MS. Due to the monomodality and monodispersity of the samples, PTA could be used without limitations [48].

**Table 6:** Particle tracking analysis (PTA) results for a given number of runs *n*

	Number concentration *C* ($n = 10-15$)			Particle size *d* ($n = 10-15$)		
Sample	*C* /$\textrm{kg}^{-1}$	*U*(*C*) /$\textrm{kg}^{-1}$	REU /$\%$	*d* /nm	*U*(*d*) /nm	REU /$\%$
04-HOB-SNP082	$1.80 \times 10^{14}$	$4.10 \times 10^{13}$	23	242.1	9.8	4.1
09-HOB-K400-05	$7.88 \times 10^{13}$	$2.19 \times 10^{13}$	28	364.4	47.4	13.0
11-HOB-AN400	$4.10 \times 10^{13}$	$8.8 \times 10^{12}$	22	424.6	18.8	4.4

REU: relative expanded uncertainty

Note: Sample 02-HOB-AN200 was not suitable for PTA measurements because it was not possible to separate particles from background and track them effectively. $U(\cdot )$ is the expanded uncertainty ($k=2$)

### Final reference values

**Fig. 6:**
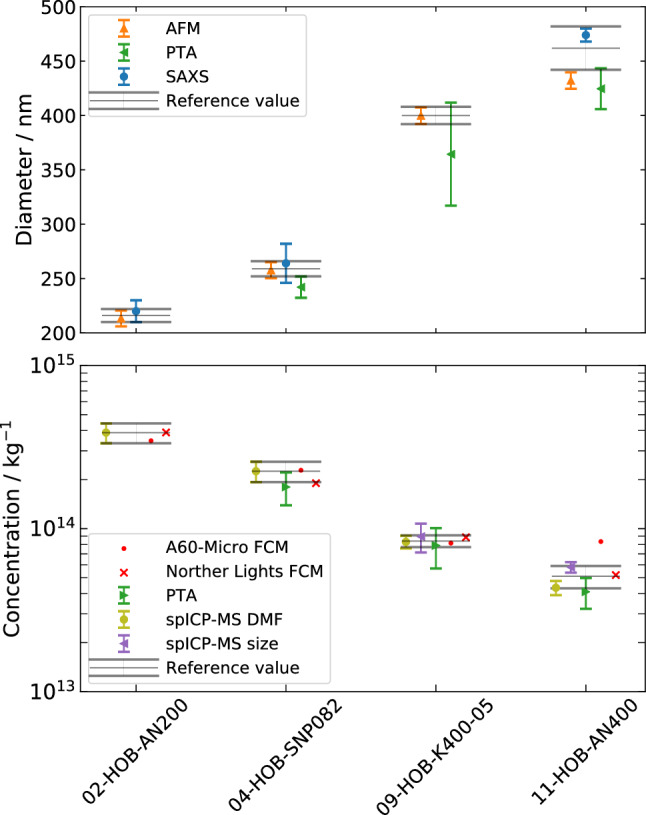
Final results of the mean diameter and number concentration of the hollow organosilica beads (HOBs) including traceable reference values. The reference values for HOB diameters were determined using small angle X-ray scattering (SAXS) and atomic force microscopy (AFM). The reference values for number concentrations were determined using two independent measurements by single particle mass spectrometry (spICP-MS) ("size method" vs. dynanmic mass flow (DMF)). Particle tracking analysis (PTA) and flow cytometry (FCM) results are presented for comparison

Traceable reference values for HOB diameters based on SAXS and AFM measurements were combined using the Gaussian uncertainty propagation. The reference value of the 09-HOB-K400-05 diameter is solely based on the AFM measurement. The reference values for the number concentration of the HOB samples are combined from two spICPMS measurements ("size method" vs. DMF). It is assumed that the two spICPMS results were completely uncorrelated. For the largest particles (11-HOB-AN400), the mean and uncertainty values were inconsistent and the Birge ratio method was used to combine the inconsistent values with a Birge ratio of 4.55.

Table 7 and Fig. 6 show all reference values, PTA results, and FCM results. With the exception of 11-HOB-AN400 measured with Apogee A60-Micro, the FCM results are all within the range of the corresponding traceable reference values.

**Table 7:** Reference values for the mean diameter *d*, the width of the size distribution $\sigma _{d}$, and the number concentration *C*

	Reference values			FCM		PTA	
Sample	$d \pm U(d)$ /nm	$\sigma _{d}$ /nm	$C \pm U(C)$ /$\textrm{kg}^{-1}$	*C* /$\textrm{kg}^{-1}$	*C* /$\textrm{kg}^{-1}$	$d \pm U(d)$ /nm	$C \pm U(C)$ /$\textrm{kg}^{-1}$
02-HOB-AN200	$216 \pm 6$	4	($3.9 \pm 0.6$)$\times 10^{14}$	$3.46$ $\times 10^{14}$	$3.89$ $\times 10^{14}$		
04-HOB-SNP082	$259 \pm 7$	10	($2.3 \pm 0.4$)$\times 10^{14}$	$2.28$ $\times 10^{14}$	$1.90$ $\times 10^{14}$	$242.1 \pm 9.8$	($1.80 \pm 0.41$) $\times 10^{14}$
09-HOB-K400-05	$400 \pm 8$	45	($8.4 \pm 0.7$)$\times 10^{13}$	$8.15$ $\times 10^{13}$	$8.85$ $\times 10^{13}$	$364.4 \pm 47.4$	($7.88 \pm 2.19$) $\times 10^{13}$
11-HOB-AN400	$462 \pm 20$	28	($5.1 \pm 0.8$)$\times 10^{13}$	$8.33$ $\times 10^{13}$	$5.19$ $\times 10^{13}$	$424.6\pm 18.8$	($4.10 \pm 0.88$) $\times 10^{13}$

FCM: flow cytometry; PTA: particle tracking analysis

The reported uncertainties *U* correspond to a confidence level of $95\%$ ($k=2$). For the concentration values referring to FCM, the first column refers to the measurement with the Apogee A60-Micro, while the second column refers to the measurement with the Cytek Northern Lights. For the concentration values, the first column refers to the measurement with the Apogee A60-Micro, while the second column refers to the measurement with the Cytek Northern Lights

## Conclusion

EVs serve as promising biomarkers for various diseases in terms of their size and number concentration [49]. In this study, we characterized four different types of HOBs that serve as potential RMs for optical measurements of the number concentration of suspended EVs by optical techniques such as FCM or PTA, which are widely used in clinical applications due to their rapid, single particle detection. Since calibration is the main challenge in the FCM analysis of EVs, well-described RMs are needed to translate the measured light scattering within defined size gates from arbitrary units to absolute units of number concentration. To ensure that the HOB samples presented have similar size (i.e. diameter), shape and optical properties to EVs when measured with FCM, we first determined traceable values for their size distribution and number concentration, including uncertainty estimates.

AFM and SAXS were used to obtain reference values for the size distribution, while SAXS also provided an uncertainty estimate for shell thickness with limit values. The mean particle size and uncertainty estimate of shell thickness were used to calculate the corresponding interval of shell refractive index for each HOB sample with FCM. Realistic RI values, i.e., values corresponding to those of EVs of similar size, were found especially when the upper limits of the corresponding shell thickness were used for the calculation. For 11-HOB-AN400, the effective RI was additionally calculated, which is in good agreement with the mean effective RI of EVs in human urine.

In addition, the number concentration was determined using two independent FCMs. With the exception of one FCM measurement of 11-HOB-AN400, all concentration values agree with the reference values within the uncertainty ranges determined by spICP-MS. spICP-MS has the advantage here that, unlike SAXS and other traceable measurement methods, it does not require additional information about the mass density or RI of the HOB shell to measure the number concentration in absolute units.

Overall, the selected HOBs in the size range of $200 \, \textrm{nm}$ to $500 \, \textrm{nm}$ were found to be EV-like in terms of their optical properties for EVs with a comparable size. Moreover, the effective RI value of HOBs can be tuned by adjusting the shell thickness, making them more suitable than polystyrene beads for application-oriented medical calibration of FCM in the future. Since EVs in human body fluids have a broad size distribution with fractions over several orders of magnitude, RMs for FCM calibration of additional size gates, including HOBs, remain to be explored. This is especially true for the size range below $200 \, \textrm{nm}$, as the sensitivity of some modern FCM devices has recently increased, so that HOBs in the size range from $100 \, \textrm{nm}$ to $200 \, \textrm{nm}$ could become the focus of future metrology projects.

## Data Availability

Data can be provided by the corresponding author upon request.
